# A commentary on establishing a local centre of excellence for research and training in pharmacometrics: Lessons from the pharmacometrics Africa–Uganda chapter

**DOI:** 10.1111/bcp.15832

**Published:** 2023-07-07

**Authors:** Letisha Najjemba, Aida Nakayiwa Kawuma, Francis Williams Ojara, Bonniface Obura, Christine Sekaggya‐Wiltshire, Walter Arinaitwe, Ruth Kikonyogo, Jackson Mukonzo, Goonaseelan (Colin) Pillai, Catriona Waitt

**Affiliations:** ^1^ Infectious Diseases Institute, College of Health Sciences Makerere University Kampala Uganda; ^2^ Department of Pharmacology and Therapeutics Gulu University Gulu Uganda; ^3^ Department of Pharmacology and Therapeutics University of Liverpool Liverpool UK; ^4^ Department of Pharmacology and Therapeutics Lira University Lira Uganda; ^5^ Department of Medicine, College of Health Sciences Makerere University Kampala Uganda; ^6^ Department of Pharmacology and Therapeutics, College of Health Sciences Makerere University Kampala Uganda; ^7^ CP+ Associates GmbH Basel Switzerland; ^8^ Division of Clinical Pharmacology University of Cape Town Cape Town South Africa; ^9^ Pharmacometrics Africa Cape Town South Africa

**Keywords:** clinical pharmacology, mentorship, pharmacodynamic, pharmacokinetic, pharmacometrics, supervision, training

## 1 INTRODUCTION

Pharmacometrics involves the development and application of mathematical models, informed by knowledge on pharmacology, physiology and the disease process, to characterize the interactions between drugs and patients. It is applied to studying pharmacokinetics, pharmacodynamics and disease progression. The ethical necessity to study drugs in the populations where they are to be used is increasingly recognized. To maximize the value of clinical data, study design and analysis must be appropriate. Historically, many datasets have been sent from African countries to better‐resourced settings for analysis, but in recent years, it has been demonstrated that capacity can be built and sustained within low‐ and middle‐income countries (LMIC)^1^ We report the establishment of the Ugandan Chapter of Pharmacometrics Africa, to share learning with other countries seeking to build similar capabilities.

Current pharmacometric approaches require computational power, appropriate software and knowledge of the mathematical principles and assumptions underpinning the techniques. Between 2012 and 2016, academic‐industry collaborations were established between Makerere University and several US pharmacometricians. This principally comprised the delivery of workshops in Uganda. Recognizing the need to both build and sustain capacity in these skills across the continent, Pharmacometrics Africa (www.pmxafrica.org) was launched in 2018, to develop quantitative clinical pharmacology among African scientists and more generally among scientists in LMIC. Pharmacometrics Africa provides online training courses, hands‐on workshops, mentorship and supervision of scientists conducting projects that use quantitative methods addressing locally relevant healthcare questions. All training materials from Pharmacometrics Africa are provided under Creative Commons licence arrangements.

The Infectious Diseases Institute (IDI), Makerere University, was established in 2002 in Kampala, Uganda, by the Academic Alliance for AIDS Care and Prevention in Africa.^2^ The IDI has since built international strength in clinical research surrounding the treatment of infectious diseases in Africa.

Despite the growing awareness of pharmacometrics as a discipline, and increasing numbers of individuals possessing basic and intermediate skills, until about 2016, almost all pharmacokinetic data generated within Uganda were exported to collaboration partners for analysis. This was necessary because, despite an increasing number of individuals having undergone training in the techniques, there was not yet a functional group of pharmacometricians capable of undertaking complex analyses with confidence.

From 2019, Pharmacometrics Africa expanded from local in‐person training sessions to host online introductory courses on pharmacology and pharmacometrics.

In parallel, IDI established expertise in online teaching and training (https://elearning.idi.co.ug/) using the Moodle platform, for a variety of clinical topics. Despite hosting the Pharmacometrics Africa online courses between 2019 and 2021, there was a heavy reliance on international tutors. However, an increasing number of Ugandan researchers had accessed training and mentorship through Pharmacometrics Africa and continued to work within the discipline to the point where there was a ‘critical mass’ of skills within Uganda. Therefore, in 2021, we officially launched the Ugandan Chapter of Pharmacometrics Africa comprising of individuals trained in pharmacometrics but working in Uganda with the aim to

1. Transfer the primary training responsibility to local faculty for sustainability.
2. Build upon regional strengths in pharmacometrics to establish a local centre of excellence.
3. Attract increasing collaborations on quantitative clinical pharmacology projects.
4. Enhance the credibility of pharmacometrics in research, regulatory, and clinical practice among Ugandan stakeholders.

In line with the first aim of the chapter, from September to December 2021, the Ugandan Chapter hosted the first clinical pharmacology course led by an all‐Ugandan team of tutors. Preparations involved consolidating the team of tutors, allocating roles and reviewing existing course content. Because previous iterations of the 12‐week online pharmacometrics course had been hosted by IDI, it was relatively straightforward for the team to update these resources for a subsequent, locally led iteration.

While the online course was to be the first specific activity of the Ugandan Chapter, the process of team building, content review and consideration of teaching skills and styles also provided the opportunity to launch the Ugandan Community of Practice in Pharmacometrics. Throughout this process, senior members of the Pharmacometrics African team (Colin Pillai, Paolo Denti and Leon Aarons) provided mentorship and ‘train the trainers’ style coaching.

The course was advertised among our networks, mailing lists and via social media to reach potentially eligible individuals. Candidates were required to show a background of proven numerical ability and planned use of pharmacometrics in their work. Priority was given to those from within Africa, although international applications were welcomed. A version of this course is available on the IDI e ‐earning website (https://elearning.idi.co.ug/index.php/courses/).

We received 247 applications and invited 42 candidates to participate. A total of 18 (43%) participants were from Uganda, seven from Nigeria, two from Brazil and India, three from Tanzania and South Africa and one each from Kuwait and Sierra Leonne. Their fields included biochemistry, biological sciences, biotechnology, clinical epidemiology and public health, laboratory medicine, medicine, pharmacology, mathematics, midwifery and pharmacy. The course was facilitated by seven Ugandan faculty members and four guest tutors who are all Africans with previous Pharmacometrics Africa experience.

Of the 42 invited candidates, five did not start. Consequently, the course was attended by 37 participants from eight countries. Of the 37 participants, a further 5 withdrew during the course primarily due to competing demands on their time, 6 did not complete the course due to failure to complete the course tasks and 26 (71%) met the criteria for the award of a certificate of completion. The criteria for completion included completion of at least 70% of the tasks, consistently logging onto the online learning platform and engagement in discussions. To enhance participants' engagement, participants were placed into groups of about five, each with a mentor (one of the tutors) who offered guidance weekly. The assessment focused on identifying potential and interest in the discipline rather than on achieving a specific grade. Participants were granted extra time to complete tasks and engage with course materials. We adapted the course programme to include an additional ‘consolidation week’ between weeks 9 and 10 to enable participants to catch up and spend time working on the practical exercises with support from the tutors. Weekly feedback was almost universally positive, but the biggest area of concern reported by the students was the time required to complete the self‐study materials and truly engage with the hands‐on exercises. Table 1 below shows the lessons learnt and how this initial activity consolidated the creation of the Ugandan Community of Practice in Pharmacometrics.

**TABLE 1:** Lessons learnt and how the Ugandan Chapter has consolidated the community of practice in pharmacometrics in Uganda.

Lessons learnt
Congregating factor: Establishment of a group needs to be motivated by a strong reason for people to work together. Organizing the course brought together several stakeholders and ignited further passion to work collaboratively.
2 There is a high but unmet demand for pharmacometrics training in Uganda: Most participants were students from academia. There is an unmet need for further scholarship opportunities (Masters and PhD level) within Uganda.
3 Multimodal and multimedia interactions via platforms like WhatsApp, Zoom meetings and the interactive online course platform enabled better interactions and sharing of ideas. Many of these interactions continued beyond completion of the course.
4 Hierarchical and peer‐to‐peer mentorship frameworks. Senior pharmacometricians need to mentor junior pharmacometricians, and the junior pharmacometricians need to mentor the junior scholars. Peer mentorship between colleagues working at a similar level brings additional benefits in mastering and applying complex skills.
5 Need for practical, hands‐on support. Sessions that were most challenging for students were those which comprised practical examples rather than theory alone. Through virtual platforms, hands‐on support was provided, and a similar virtual hands‐on approach forms the basis of ongoing ‘lab meetings’.

The need for universities in LMIC to develop graduate‐level pharmacometrics programs has been well‐demonstrated. Pharmacology is embedded within the medical and pharmacy curricular of all approved medical and graduate programs in Uganda. Makerere University, Mbarara University and Kampala International University all have MSc in pharmacology training programs that have huge components of clinical pharmacology, but they may be reluctant to invest the time and money required to hire experts in the field as faculty and to support them until external funding can be established. In some cases, this is because of lack of understanding of the potential role of pharmacometrics in addressing key health priorities within the population. In addition to raising awareness of pharmacometrics as a key discipline to guide safe, effective use of medication in a population, there is a need for increased donor support for pharmacometrics scholars and fellows. Academic–industry partnership and placements of junior scholars in established working groups will facilitate further mentorship and build broader expertise. Through such efforts, pharmacometrics can indeed mature and grow as a scientific discipline on the African continent. The regional hub provides a platform to collaborate with other universities such as University of Cape Town (UCT) in South Africa, Strathmore University in Kenya among others. The hub can now train other pharmacometricians where in the past, only UCT was available.

In the year since the inception of the Ugandan Chapter, several priorities to increase pharmacometrics research in Uganda have been established, as summarized in Table 2.

**TABLE 2:** Measure to increase pharmacometrics research in Uganda.

Perceived gap	Examples, comments and proposals
Skills training, hands‐on experience and certification	Postgraduate training programs, for example, a Masters in Pharmacometrics. These will need contextualization to Africa–Ugandan healthcare needs and should use a combination of available and affordable state‐of‐the‐art distance learning pedagogy as popularized by Coursera (https://www.coursera.org/) and traditional classroom teaching.
Accessibility of high‐quality, locally generated datasets via online repositories and collections	Adoption of standards to support best practices in making data Findable, Accessible, Interoperable and Reusable (FAIR).
Clinical trials capacity	Increasing academic‐industry collaboration to enable earlier phase clinical trials in diseases affecting African populations.
Infrastructure and environment	Develop an accessible network of peers and mentors for scientific collaboration including soft skills, mentorship and coaching. Provide access to high‐speed computing clusters with relevant software for data analysis and simulation including software, hardware and bandwidth.
Advocacy and translation to benefit healthcare	Funding agencies should recognize the high benefit *vs*. smaller relative investment inherent in pharmacometrics research *vs*. the laboratory‐based sciences. The need for us to advocate, educate and empower. This should ultimately translate into national and local treatment guidelines, algorithms and standards of care.
Stronger industry partnerships most especially in PBPK which provides a mechanistic understanding of drug disposition in populations with limited data	The priority of PBPK research of industry investment for earlier phase trials and internships that could be in both directions. Adoption of standards for the transparent reporting and data sharing from existing work and where such standards might not exist, to define them.

We currently seek to raise awareness among different potential stakeholders in pharmacometrics across Uganda. These include academic and industry partners involved in pharmacology research in Uganda, together with the ethics committees and regulatory bodies who are gatekeepers on protocols involving pharmacometrics. The Ugandan Chapter of Pharmacometrics Africa will enhance knowledge sharing, support training and mentorship and enable better advocacy for pharmacometrics research to inform dosing for local patients and with global impact.

We have demonstrated that it is possible to deliver an online course with a relatively young faculty team in Africa using an online platform. Establishing regional hubs for pharmacometrics training and collaboration will enable the development of strong, interactive partnerships, establishment of a vibrant research environment for the next generation of scientists and enable the sustained implementation of capabilities to address regional priorities in LMIC.
